# Influence of the Chungkookjang on histamine-induced wheal and flare skin response: a randomized, double-blind, placebo controlled trial

**DOI:** 10.1186/1472-6882-11-125

**Published:** 2011-12-05

**Authors:** Dae-Young Kwon, Hye-Jeong Yang, Min-Jeong Kim, Hee-Joo Kang, Hyun-Jin Kim, Ki-Chan Ha, Hyang-Im Back, Sun-Young Kim, Eun-Ok Park, Min-Gul Kim, Seok-Kwon Yun, Soo-Wan Chae, Back-Hwan Cho

**Affiliations:** 1 Clinical Trial Center for Functional Foods of Chonbuk National University Hospital, Keumam-dong, Deokjin-gu, Jeonju City, 561-712, Republic of Korea; 2 Department of Medical Nutrition Therapy, Chonbuk National University, Keumam-dong, Deokjin-gu, Jeonju City, 561-180, Republic of Korea; 3 Korea Food Research Institute, Baekhyeon-dong, Bundang-gu, Seongnam-City, Gyeonggi-do, 463-746, Republic of Korea

## Abstract

**Abstracts:**

**Background**

Allergic disease is a consequence of exposure to normally innocuous substances that elicit the activation of mast cells. Mast-cell-mediated allergic response is involved in many diseases such as anaphylaxis, urticaria, allergic rhinitis, asthma and allergic dermatitis. The development of food products for the prevention of allergic disease is an important subject in human health. The chungkookjang (CKJ) has been reported to exhibit antiallergic inflammatory activity. Therefore, the aim of the study is to examine the effects of the CKJ to reduce histamine-induced wheal and flare skin responses.

**Methods/Design**

A randomized, double-blind, placebo-controlled study in 60 healthy subjects will be carried out. Sixty volunteers (aged 20-80) who gave a written consent before entering the study will be randomized in two groups of thirty subjects each. The skin prick test with histamine solution of 10 mg/ml will be performed on the ventral forearm, 10 cm from the elbow. The subjects will be instructed to take 35 g per day of either the CKJ pills or a placebo pills for a period of 3 months. Diameters of wheal and flare will be assessing 15 minutes after performing the above-mentioned skin prick test. The primary outcome is change in wheal and flare responses. Secondary outcomes will be include change in serum histamine, immunoglobulin E, cytokines (interferon-gamma, interleukin-4, -10, and tumor necrosis factor-alpha), and eosinophil cationic protein.

**Discussion**

This study will show the potential anti-inflammatory properties of the CKJ in their skin activity when histamine is the challenging agent as occurs in the clinical situation. And the present protocol will confirm the efficacy and safety of the CKJ for allergy symptoms, suggesting more basic knowledge to conduct further randomized controlled trials (RCT). If this study will be successfully performed, the CKJ will be an alternative dietary supplemental remedy for allergy patients.

**Trial Registration**

NCT01402141

## Background

Allergic reaction is a hypersensitive response caused by immunoglobulin E (IgE)-dependent mast cell activation. In response to IgE receptor cross-linking or other stimuli, mast cells initiate exocytosis of the contents of secretory granules. These include vasoactive amines, arachidonic acid metabolites, chemokines, and cytokines [1,2]. These pro-inflammatory mediators are responsible for activating mast cells in allergic inflammation and the hypersensitive response [3-6]. Therefore, an agent or agents capable of attenuating the production of these mediators may be used as anti-inflammatory/anti-allergic agents. Since the notion that a daily intake of certain food having anti-allergic activity has the potential to reduce or eliminate the need for drugs is generally accepted, the anti-inflammatory/anti-allergic activities of the chungkookjang (CKJ) have been screened for this purpose [7].

Soybean-based fermented foods, such as CKJ is a Korean traditional food. It is prepared by fermenting unsalted soybeans with *Bacillus subtilis *for several days. It has a long history of popular use by the Korean people and is sometimes used for topically for inflammatory skin disorders. Several isoflavonoids such as genistein and daidzein have been identified among the bioactive compounds in the CKJ [8]. During the fermentation period, the content of the flavonoid aglycones increases because of hydrolysis of the flavonoid glycosides [9]. The CKJ is also containing the poly-gamma-glutamic acid (gamma-PGA). Gamma-PGA is an anionic polymer that is composed of D- and L-glutamic acid units connected by gamma-amide linkages between alpha-amino and gamma-carboxylic acid groups [10]. Gamma-PGA is mainly produced by *Bacillus subtilis *during fermentation processes of soybeans and not present in humans [11]. Gamma-PGA is water soluble, biodegradable, edible and non-toxic properties, raising interest in a broad range of industries including food and cosmetics [12]. Furthermore, recent reports have indicated anti-allergic activity and immune modulatory activities of gamma-PGA [7,13-16].

Histamine is one of the important mediators that elicit a variety of symptoms such as wheal and flare response. Since these histamine-related symptoms provoke the urge to scratch, often impairing the quality of life, measures to suppress and control these symptoms are required. In the development of a new anti-histamine, the histamine-induced wheal and flare skin test has been used most frequently. The histamine-induced wheal and flare test is based on the measurement of local swelling caused by plasma extravasation (wheal) and reflex vasodilatation (flare) after histamine challenge [17]. Therefore, the histamine skin prick test may be useful for assessing aspects of drug activity during the early clinical development of a new anti-histamine.

The aim of this study is to evaluate the efficacy and safety of the CKJ, a dietary supplement for the treatment of allergic skin symptoms. This study will be first pilot study about the improvement of skin hypersensitivity reaction of the CKJ in healthy volunteers. The results of this study will give us the clinical of biochemical parameters such as IgE, histamine, and cytokines between the placebo and CKJ groups. On the basis of the results, we will suggest the optimal design, precise sample size, and primary outcome for a further large scale RCT.

## Methods/design

### Study objectives

The objectives of this RCT are to study whether the CKJ pills can alleviate the histamine-induced wheal and flare skin responses in healthy subjects.

#### Primary objective

To evaluate the efficacy of the CKJ pills on histamine-induced wheal and flare skin responses in healthy subjects after 12 weeks of consumption.

#### Secondary objectives

To evaluate the following factors in healthy subjects after 12 weeks of consumption on:

A) Change of IgE and histamine concentrations

B) Change of cytokines such as IFN-gamma, IL-4, IL-10, and TNF-alpha

C) Change of eosinophil cationic protein

### Ethics

The study protocol and the written informed consent were approved by the Functional Foods Institutional Review Board of Chonbuk National University Hospital (CUH IRB 2010-02-007). Each participant will be notified regarding the study protocol. Written informed consent will be obtained from each participant.

### Study/trial design

**Figure 1:**
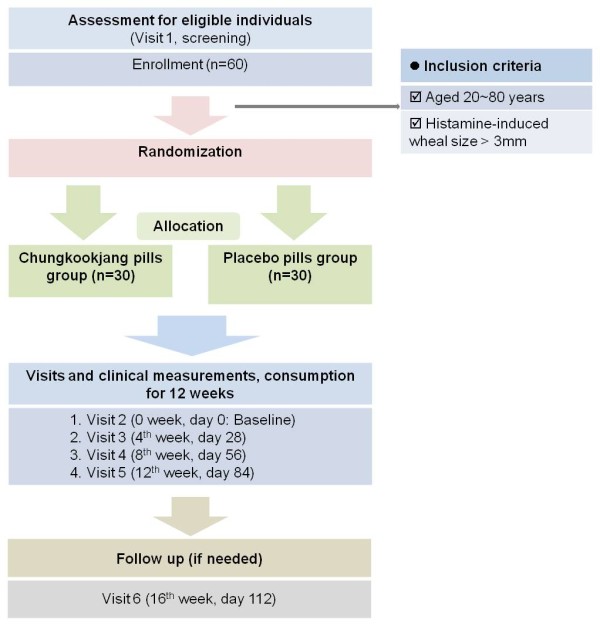
**Flow diagram for study**. Summary of the study flow.

This study is a randomized, double-blind, placebo-controlled, two-armed parallel clinical trial, comparing the CKJ pills to placebo pills. The design of the study will integrate rigorous in accord with principles set out in the Declaration of Helsinki and the Good Clinical Practice guidelines. Our study plan is summarized in Figure 1.

### Inclusion criteria for participation in the trial

#### Inclusion criteria

Participants will be included if they meet the following criteria:

A) Healthy volunteers aged 20 to 80 years

B) Positive response to histamine skin prick test(a wheal size > 3 mm)

C) Ability to give informed consent

#### Exclusion criteria

Participants will be excluded if they:

A) have severe generalized skin conditions such as eczema, psoriasis, and atopy(hay fever, asthma)

B) have severe allergic reactions in the past

C) have taken oral anti-histamines or topical corticosteroids in the preceding 3 months

D) have any acute or chronic illness

E) have cardiovascular disease, liver or kidney disease

F) have allergies on soybean foods

G) have taken any prescribed or investigation medication during the 8 weeks preceding enrolment.

H) have history of drug or alcohol abuse in prior 2 months

### Recruitment

60 participants will be recruited through local advertising and doctor referrals from hospital outpatients and general practice clinics. Interested participants can telephone or email the trial coordinators at the trial conducting sites for further information. Participant information and consent forms will be sent to interested individuals to read over prior to scheduling their first visit.

### Randomization

After enrollment, participants will be randomly assigned to one of the two groups, either the CKJ pills group or placebo pills group. The allocation ratio will be 1:1 in blocks of 2. Randomization will be performed at a site remote from trial location. Random numbers will be generated by a computerized random-number generator through the block-randomization method of a software program (Excel, Microsoft Office 2007) for sequence generation. At the time of randomization participants will draw an envelope. Each envelope contains a number that is concealed to the treatment allocation. Randomization sequence and allocation will be concealed to all study subjects, research staff, investigators and pharmacists until completion of the study. The allocation list will be protected by password access files and held by a non-investigator independent. In the event of an emergency medical situation the individual's randomization code and group allocation can be identify.

### Outcome measures

#### Primary outcome

The primary outcome is the difference on wheal and flare skin responses induced by histamine challenge at baseline and three months after randomization. The histamine-induced wheal and flare skin test will be performed by histamine challenge via epicutaneous prick. The resulting wheal is a sign of the direct vascular effects of histamine, namely increased vascular permeability and skin blood flow, while the flare reflects vasodilatation due to histamine-induced reflex neuropeptide release from local nerve terminals. The wheal and flare response will be assessed 15 min after the test and will be assessed as the mean diameter or by planimetry.

#### Secondary outcomes

Secondary outcomes will be shown by the mean change of IgE, histamine, eosinophil cationic protein, IFN-gamma, IL-4, IL-10, and TNF-alpha. These biomarkers will be checked at weeks 0 (baseline) and 12 (end of the trial). The schedule of assessments is presented in Table 1.

**Table 1:** A brief study schedule at every visit

	*Screening Visit 1*	*Baseline Visit 2*	*Visit 3*	*Visit 4*	*Visit 5*	*Follow up (Visit 6)*
	
	*D-21 ~D-1*	*Week 0 D0*	*Week 4 D28*	*Week 8 D56*	*Week 12 D84*	*Week 16 D112*
***Informed consent form***	◯					

***Demographic information taking***^***1***^	◯					

***Medical history taking***	◯					

***Inclusion/exclusion criteria check***	◯	◯				

***Physician examination***^***2***^	◯				◯	

***Drinking/smoking taking status***^***3***^	◯				◯	

***Vital sign measurement***	◯	◯	◯	◯	◯	

***Concomitant drugs check***	◯	◯	◯	◯	◯	

***Electrocardiogram(ECG)***	◯				◯	

***Histamine skin prick test***	◯				◯	

***Laboratory test***^***4***^	◯				◯	

***Histamine & ECP***		◯			◯	

***Study product distribution***		◯	◯	◯		

***Compliance checking***			◯	◯	◯	

***Adverse event monitoring***			◯	◯	◯	◯

***Diet, physical exercise counseling***^***5***^		◯	◯	◯		

◯ *: Item need to be performed during the visit*

^1 ^*Sex, date of birth, age, contact address and phone number*

^2 ^*Include present illness, past history taking*

^3,5 ^*Risk factors such as alcohol drinking, smoking, severe exercise and other dietary supplement intake will be managed*

^4 ^*Blood test(WBC, RBC, hemoglobin, hematocrit, platelets count, AST, ALT, ALP, gamma-GTP, albumin, BUN, creatinine, glucose, total bilirubin, total protein, total-cholesterol, triglyceride, LDL-C, HDL-C, IgE), urine test(specific gravity, pH, Protein, Ketone, Glucose, bilirubin, urobilinogen, nitrite, microscopic (RBC, WBC))*

### Statistical analysis

#### Baseline data and Outcomes data

Statistical analysis will be performed using SAS version 9.0 for Windows (SAS Institute, Cary, NC, USA). Data will be presented as mean±SD values. The Chi-square test will be performed to determine differences at baseline in frequencies of categorized variables between the groups. A linear mixed-effects model will be applied to repeated-measures data for each continuous outcome variable. Fixed effects will be included treatment group, treatment visit, and interaction between treatment group and visit. When the analysis of variance indicated significant differences among groups, *post hoc *test (Turkey's test) will be used to separate the differences between groups before and after the 12-week intervention period. A value of p < 0.05 will be considered statistically significant.

#### Adverse events and monitoring safety

All unexpected adverse events related to CKJ pills intake will be reported to the investigator by participants and write on the individual case report form by the investigator. Safety will be assessed by the reporting of clinical laboratory tests, vital sign measurements, and adverse events. Clinical laboratory tests, including AST/ALT, BUN/creatinine, red blood cell (RBC) count, white blood cell (WBC) count, hemoglobin, hematocrit, number of platelets, and number of differentiated cells will be determined at weeks 0 (baseline) and 12 (end of the trial). Vital signs of each participant will be checked with monitoring of adverse events (nausea/vomiting, fatigue, allergic reaction, and any adverse events related to CKJ pills) after each visit.

#### Compliance

The CKJ pills remaining after each visit will be quantified in order to enhance medication compliance. Participants whose compliance with the CKJ pills is ≤ 70% of the total dose will be considered to have dropped-out.

#### Sample size

Sample size calculation is performed; primary outcome measure will be the histamine-induced wheal size, whereby 5.0 square millimeters is assumed a clinically relevant difference between the two groups, with a standard deviation of 6.85, alpha set on 5% and power on 80%. This resulted in a required number of 24 subjects in each group. Assuming a dropout rate of 20%, a total of 60 subjects will be enrolled in this trial.

## Discussion

The use of histamine-induced wheal and flare skin responses to demonstrate the antihistamine activity is a widely accepted and well established method.

The aim of this study is to evaluate the antihistamine effect of the CKJ on the skin quantitatively. For this purpose, the ability of the CKJ to suppress the histamine-induced wheal and flare skin responses will be assessed in 60 healthy volunteers.

In this study, we expect that the CKJ effectively suppress the wheal and flare skin responses. Many studies have assessed the relative potency of antihistamines by measuring their ability to suppress the histamine-induced wheal and flare skin responses. To the best of our knowledge, there has been no report demonstrating the CKJ. Therefore, if this study will be successfully performed, the CKJ may offer beneficial effects in preventing the various allergy symptoms. And these results will have important implications for the design of sustainable cost-effective health services for people with mild allergic diseases. In addition, this study will be the groundwork for the larger scale RCT. To draw confirmative conclusion about the therapeutic efficacy and safety of the CKJ, a full-scale RCT will be performed.

## Competing interests

The authors declare that they have no competing interests.

## Authors' contributions

DYK, SWC and SWC received the research funding, led the entire study, and drafted the manuscript. HJY, KCH, HIB, SYK, EOP, MJK, HJK, HJK, and SKY participated in the design of the study and performed the statistical analysis. All authors read and approved the final manuscript.

## Pre-publication history

The pre-publication history for this paper can be accessed here:

http://www.biomedcentral.com/1472-6882/11/125/prepub
